# Vocal communication in a complex multi-level society: constrained acoustic structure and flexible call usage in Guinea baboons

**DOI:** 10.1186/1742-9994-10-58

**Published:** 2013-09-23

**Authors:** Peter Maciej, Ibrahima Ndao, Kurt Hammerschmidt, Julia Fischer

**Affiliations:** 1 Cognitive Ethology Laboratory, German Primate Center, Kellnerweg 4, 37077, Göttingen, Germany; 2 Courant Research Center “Evolution of Social Behaviour”, Göttingen 37077, Germany; 3 Direction de Park National de Niokolo Koba, Tambacounda, Senegal

**Keywords:** Evolution, Vocal communication, Call structure, Call usage, Guinea baboon, Social complexity, Competition

## Abstract

**Background:**

To understand the evolution of acoustic communication in animals, it is important to distinguish between the structure and the usage of vocal signals, since both aspects are subject to different constraints. In terrestrial mammals, the structure of calls is largely innate, while individuals have a greater ability to actively initiate or withhold calls. In closely related taxa, one would therefore predict a higher flexibility in call usage compared to call structure. In the present study, we investigated the vocal repertoire of free living Guinea baboons (*Papio papio*) and examined the structure and usage of the animals’ vocal signals. Guinea baboons live in a complex multi-level social organization and exhibit a largely tolerant and affiliative social style, contrary to most other baboon taxa. To classify the vocal repertoire of male and female Guinea baboons, cluster analyses were used and focal observations were conducted to assess the usage of vocal signals in the particular contexts.

**Results:**

In general, the vocal repertoire of Guinea baboons largely corresponded to the vocal repertoire other baboon taxa. The usage of calls, however, differed considerably from other baboon taxa and corresponded with the specific characteristics of the Guinea baboons’ social behaviour. While Guinea baboons showed a diminished usage of contest and display vocalizations (a common pattern observed in chacma baboons), they frequently used vocal signals during affiliative and greeting interactions.

**Conclusions:**

Our study shows that the call structure of primates is largely unaffected by the species’ social system (including grouping patterns and social interactions), while the usage of calls can be more flexibly adjusted, reflecting the quality of social interactions of the individuals. Our results support the view that the primary function of social signals is to regulate social interactions, and therefore the degree of competition and cooperation may be more important to explain variation in call usage than grouping patterns or group size.

## Background

The signal design of animal calls and the resulting morphology of their vocal repertoire is assumed to be shaped by several factors, including phylogenetic history [1,2], habitat characteristics [3-5], receiver psychology [6-8], as well as the social system of the species [9-12], including the social organisation (grouping patterns), mating behaviour, and social structure (relationships). To understand how these factors affect vocal production it is important to distinguish between the acoustic structure of calls on the one hand and the usage of vocalizations, i.e. the rate and contexts in which calls are produced, on the other, as both may be subjected to different selective pressures and constraints [13,14]. Habitat characteristics may shape both the structure of long-distance calls, to improve transmission characteristics, as well as the timing of calls during daytime [15,16] but see [17]. Moreover, it has been hypothesized that discrete repertoires with a high number of call types are selected in arboreal animal species living under poor visibility [18-20], while graded repertoires frequently occur in terrestrial species occupying more open habitats [21,22]. Further, intra- and intersexual competition will act on the structure of quality signals [23-25], while the usage of such calls may depend on the presence of potential mates or competitors, for instance, and thus vary considerably at a short-term basis. In societies with high male competition, contest- or display-signals regularly occur during agonistic interactions [26-28]. In contrast, in species with less intense competitive relationships, such status signals appear to be less common [29,30] and animals often use a variety of appeasement signals to resolve social conflicts [31-33]. Furthermore, in primate societies with extensive affiliative relationships animals exhibit several vocal signals to facilitate friendly interactions [9,34].

What is less well understood to date is the interplay between social and vocal systems. In recent years, the idea that more complex social systems may generally lead to higher vocal complexity has attracted increasing attention [9,12,35,36]. To address this question, it is necessary to pin down social complexity more clearly. One simple measure that has been frequently used is group size [12,35]. In the case of nonhuman primates, grooming duration has also been taken as a reflection of the intensity of affiliative social relationships [12]. At the level of social relationships, a more elaborate measure would encompass the differentiation and diversity of social relationships [37,38]. Social complexity may however also be assessed at the level of the social organisation (grouping patterns), that is, whether subjects live in stable groups or in multi-level fission-fusion societies. A number of recent papers have linked the evolution of social intelligence to life in fission-fusion groups e.g. [39,40]. Given that life in a multi-level society can be conceived as more socially complex, one may expect that it also favours a higher vocal complexity.

The sound production mechanisms in terrestrial mammals are well understood. In the majority of terrestrial mammal species, the acoustic structure of calls is largely innate (but see [41]) and their vocal development does not require auditory experience [14,42-44]. For instance, congenitally deaf squirrel monkeys as well as deaf mice produce their species typical sounds [42,45]. Nevertheless, auditory input may affect vocal output to a lesser or larger degree. In some mammal species, vocal plasticity, such as vocal imitation (e.g. African elephant (*Loxodonta africana*, [46]) or vocal convergence have been described, chimpanzees (*Pan troglodytes*, [47,48]). Age-related acoustic changes are commonly attributed to maturational factors, such as growth [49], practice [50] or changes in the physiology, such as variation in hormone levels [45] (but see [13] for a limited exception).

Several studies conducted among closely related species revealed a high inter-specific concordance in numerous acoustic features [51,52] and phylogenetic analyses have shown that the degree of inter-specific vocal variability is bound by genetic relatedness [53-57]. In contrast to the acoustic structure, the usage of vocal signals is considered to be more flexible and partly under voluntary control [14,58].

In the present study, we investigate the characteristics of the vocal repertoire of adult Guinea baboons (*Papio papio*), focusing on both vocal production and call usage, in order to elucidate how their social organisation and the quality of their social relationships affects both of these aspects of their vocal communication. Guinea baboons live in a complex, multi-level social organization [59,60], which differs considerably from the stable multi-male, multi-female groups of savannah baboons (i.e., chacma baboons, *P. ursinus*; olive baboons, *P. anubis* and yellow baboons, *P. cynocephalus*) [61] as well as the male-centred harem structures reported for hamadryas baboons (*P. hamadryas*) [62]. The Guinea baboon society consists of several layers, including “parties” made up of 3–5 adult males with associated females and young. Specific parties regularly team up to form a “gang”. Gangs in a given area share an almost identical home range and although they meet only sporadically during the day, they meet regularly at sleeping sites and water holes, and may occasionally form large aggregations of more than 350 individuals [60,63,64]. Guinea baboon males maintain extensive affiliative and greeting relationships with other males [60,63,65], unlike Chacma baboon males whose relationships are characterized by fierce competition [61]. Furthermore, personal observations suggest that female social relationships are relatively weak in Guinea baboons, in contrast to the strong bonds observed in savannah baboon females [66,67]. Females are the dispersing sex, further strengthening the view that the social system (sensu Kappeler and van Schaik [68]) of this species differs considerably from that of other baboon species. To date, little was known about Guinea baboon vocal behaviour in the wild (but see [69] for a study on Guinea baboon barks and [70] for a study in captivity).

Regarding the structure of the vocal repertoire, the assumption that vocal communication in nonhuman primates (and other terrestrial mammals) is highly evolutionarily constrained generates the prediction that the structure of the Guinea baboon vocal repertoire should differ only marginally from that of other baboon taxa, and that possible differences can be largely attributed to differences in morphology. In contrast, if a more complex social organisation indeed favours a higher vocal complexity [12], Guinea baboons should exhibit a larger vocal repertoire size than other baboon taxa. Regarding call usage, and following the hypothesis that the vocal communication of a species is driven by the specifics of their social interactions (i.e. call function), we predict a pronounced rate of affiliative calls, due to their largely tolerant and affiliative social structure, while we expect a diminished occurrence of contest and display vocalizations, irrespective of the possible changes in repertoire structure.

We used two-step cluster analyses to quantitatively classify the Guinea baboon vocal repertoire. To estimate call rates we collected 190 h of focal observations from 18 subjects. In addition, we analysed the structure and occurrence of their “grunt” vocalizations in detail. In other baboon taxa, grunts have been shown to function to coordinate and mediate various interactions among group members (e.g. affiliation [71], reconciliation [72] and threat [73]). In light of the strong bonds between males, we expect that grunts play an important role in the regulation of their relationships.

## Results

### Vocal repertoire

#### Call structure

**Figure 1:**
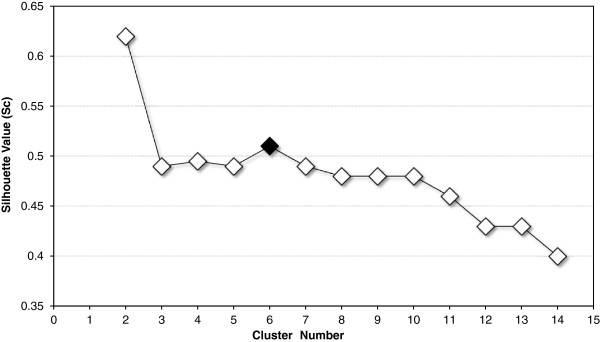
**Results of a two-step cluster analysis.** Respective Silhouette values (*Sc*) of each clustering are shown in the graph. The black dot indicates the solution chosen for the discriminant function analysis (DFA). This six call-cluster solution was the most appropriate one, since it yields a high cluster validity and corresponded to most of the audibly distinct call types.

**Figure 2:**
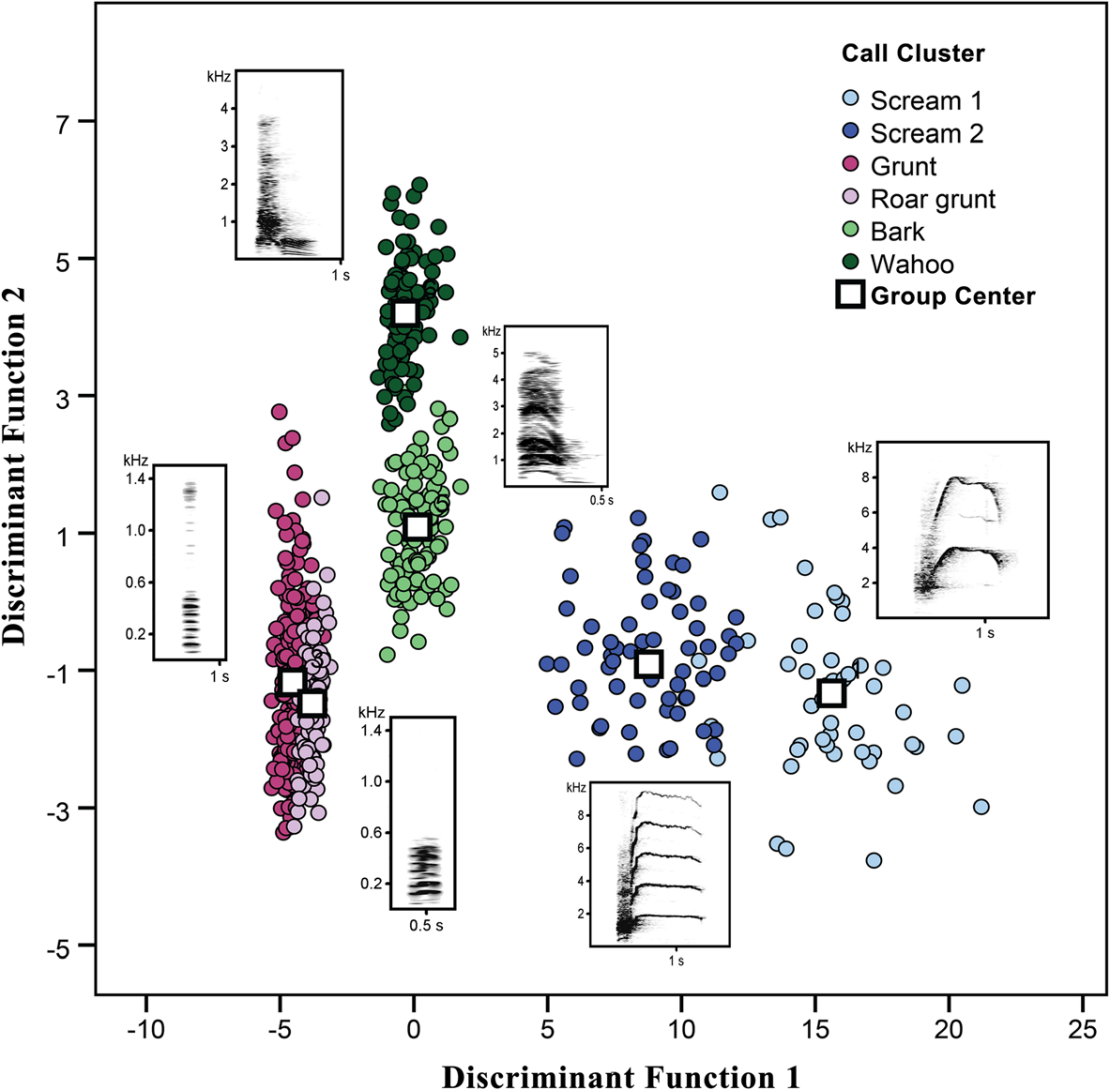
**Discriminant function analyses with the six calculated call-clusters used as grouping variable.** DFA2 mean and F0 mean had the highest load on the first discriminant function; Cs mean and Hnr1 loaded strongest on the second one. The five respective discriminant functions revealed a high overall classification success; i.e. 99.2% of calls were correctly assigned (cross-validated, step-wise DFA). For each call-cluster a representative spectrogram is shown in the figure.

The cluster solution with the highest validity (*Sc* = 0.62) contained only two clusters, one with screams and one with all other calls (see Figure 1). The next best cluster solution was the one containing six clusters (*Sc* = 0.51). A higher number of clusters did not lead to a higher validity. The comparison between the audio-visual classification and the six calculated call cluster revealed a concordance of 91%, and although screams were separated into two clusters, all other call clusters largely coincided with the audio-visual classification. Therefore, we settled on the six-cluster classification schema for further analysis and labelled the clusters by using the same onomatopoetic terms as used during studies conducted on the vocal communication of savannah baboon: screams e.g. [74], female barks e.g. [75], male wahoos (a two syllable bark e.g. [76]), grunts e.g. [77] and roar grunts e.g. [78]. All call types have been clearly distinguished by the six cluster solution. Figure 2 illustrates the differences between the clusters based on the results of the discriminant function analysis. The classification procedure indicated that the clusters could be discriminated well (99.2% correct classification, cross validated).

To discriminate between male wahoos and female barks the most important parameter was the consistency of time segments (Cs mean, see Table 1 for a detailed description of the acoustic parameters used for the analyses) within the call, which was lower in male than in female loud calls. Furthermore, the first dominant frequency band was more strongly modulated (DFB1ch mean) in male wahoos than in female barks. The screams differed mainly in the pitch parameters; where the first cluster exhibited a higher fundamental frequency (F0), a wider frequency range (Range mean) and a higher distribution of frequency amplitudes (DFA2 mean) than the second cluster. Screams uttered by males or females were structurally similar and were not distinguished by the cluster analysis. To differentiate between roar grunts and grunts the call duration (Duration) was the most important parameter followed by frequency range. Roar grunts were much longer compared to grunts and had a narrower frequency range. In Table 2 the mean values of the acoustic parameters for each call-cluster are shown.

**Table 1:** Description of the acoustic parameters used in the analysis

**Abbreviation**	**Formulation**	**Detailed description**
Duration [ms]^*^	Duration	Call duration measured within the adjusted start- and end thresholds
DFA2 mean [Hz]^*^	Distribution of frequency amplitudes	Frequency at which the distribution of frequencies reaches the second quartile of the total distribution, mean values across time segments
DFB1ch mean [Hz]^*^	Modulation of the first dominant frequency band	Mean deviation between 1st dominant frequency band and floating average curve
Range mean [Hz]^*^	Frequency range	Mean difference between the highest and the lowest frequency
PFtr mean [Hz]^*^	Trend of the peak frequency	Mean deviation between the peak frequency (PF) and the liner trend
Cs mean^*^	Consistency of time segments	Mean correlation coefficient of all successive time segments of the call
F0 mean [Hz]^+^	Fundamental frequency	Mean fundamental frequency across all time segments in which a harmonic structure could be detected
Noise [%]^+^	Noisiness	Percentage of time segments in which no harmonic or disturbed structure could be detected
Hnr1 ^+^	Harmonic to noise ratio	Harmonic to noise ratio in the first third of the overall call frequency (1 = no noise)

The general macro (^*^) as well as the manual tonal macro (^+^) of LMA 2012 was applied for parameter extraction.

**Table 2:** Mean values (Mean ± SD) of the acoustic parameters for each call-cluster and copulation calls

**Cluster**	**Scream 1**	**Scream 2**	**Grunt**	**Roar grunt**	**Bark**	**Wahoo**	**Copulation call**
	**(*N*** **= 52)**	**(*N*** **= 70)**	**(*N*** **= 226)**	**(*N*** **= 91)**	**(*N*** **= 94)**	**(*N*** **= 101)**	**(*N*** **= 25)**
Duration [ms]	1045 ± 359	876 ± 292	180 ± 34	455 ± 79	290 ± 58	319 ± 66	97 ± 20
DFA2 mean [Hz]	4460 ± 747	2685 ± 510	434 ± 102	341 ± 51	1087 ± 146	963 ± 142	1414 ± 625
DFB1ch mean [Hz]	127 ± 57	71 ± 44	9 ± 8	3 ± 2	49 ± 28	72 ± 22	80 ± 77
Range mean [Hz]	4468 ± 990	2444 ± 696	952 ± 316	530 ± 189	1606 ± 393	1722 ± 328	2137 ± 1150
PFtr mean [Hz]	870 ± 428	385 ± 239	72 ± 47	35 ± 27	163 ± 65	176 ± 59	236 ± 117
CS mean	0.97 ± 0.01	0.97 ± 0.01	0.97 ± 0.02	0.99 ± 0.01	0.97 ± 0.01	0.92 ± 0.01	0.97 ± 0.01
F0 [Hz]	2352 ± 577	1758 ± 267	80 ± 23	70 ± 10	588 ± 105	472 ± 81	290 ± 149
Noise [%]	63 ± 20	53 ± 18	14 ± 10	42 ± 14	24 ± 14	41 ± 12	47 ± 19
Hnr1	0.16 ±0.06	0.31 ± 0.08	0.26 ±0.07	0.39 ± 0.07	0.46 ±0.07	0.46 ± 0.04	0.37 ± 0.12

Copulation calls have not been statistically classified by the cluster analysis.

In general, for the overall call-cluster discrimination of the call repertoire, the most important acoustic parameters were the harmonic to noise ratio (Hnr1), the DFA2 mean and the Cs mean; whereas the trend of the peak frequency (PFtr mean) and the noise (Noise) of the call as well as the DFB1ch mean only moderate contributed to the classification procedure.

In contrast to other baboons, female copulation calls were only rarely uttered; hence, we could only qualitatively assess the acoustic structure of Guinea baboons’ copulation calls (see Table 2). Furthermore, threat calls, a common vocal pattern in savannah baboons, where extremely soft, precluding any acoustic analyses.

### Call usage

#### Bark

Females produced barks in three different contexts (see Table 3), most frequently in the Forage/Travel context ( $\bar{x}$ = 73.7 ± 20.7 %). In 61.7% of all barks uttered during Forage/Travel, visibility was middle or dense and only 5–10 animals were visible. Barks were also regularly produced during alarm situations ( $\bar{x}$ = 20.7 ± 26.1 %), mostly when subjects appeared to be threatened by the observer or by predators. Occasionally they were produced when a female was harassed by other females ( $\bar{x}$ = 5.7 ± 9.8 %).

**Table 3:** Percentage of usage of the call types (Mean ± SD) in the different contexts

**Context**	**Scream**	**Grunt**	**Roar grunt**	**Bark**	**Wahoo**
Forage/Travel		♂ 7.1 ± 3.4%	♂ 11.0 ± 15.6%	♀ 73.7 ± 20.7%	♂ 80.0 ± 22.4%
		♀ 11.2 ± 9.8%			
Alarm	♀ 8.3 ± 15.6%		♂ 5.3 ± 7.5%	♀ 20.7 ± 26.1%	♂ 11.7 ± 18.6%
Agonistic	♂ 100.0 ± 0.0%		♂ 83.3 ± 23.6%	♀ 5.7 ± 9.8%	♂ 8.3 ± 18.6%
	♀ 91.5 ± 16.0%				
Affiliation		♂ 39.3 ± 5.1%			
		♀ 48.1 ± 18.9%			
Infant handling		♂ 25.2 ± 4.5%			
		♀ 20.2 ± 8.5%			
Greeting		♂ 28.4 ± 9.5%			
		♀ 20.5 ± 19.8%			

#### Wahoo

Similarly to female barks, male wahoos were mostly produced in Forage/Travel contexts ( $\bar{x}$ = 80.0 ± 22.4 %); 62.7% of all wahoos produced during Forage/Travel were uttered when visibility was poor and only 5–10 animals were visible. Additionally, wahoos were uttered during alarm situations ( $\bar{x}$ = 11.7 ± 18.6%) and, occasionally during encounters with other gangs ( $\bar{x}$ = 8.3 ± 18.6%). Wahoos were not produced during aggressive interactions and throughout the study period we never observed males producing wahoos while chasing or attacking other males.

#### Scream

Males and females produced screams mainly during agonistic interactions. While adult females occasionally started to scream during alarm situations ( $\bar{x}$ = 8.3 ± 15.6%), adult males were never observed to produce screams under such circumstances. All male screams that we heard were produced during agonistic interactions with other males. Females screamed primarily in agonistic contexts, while they were harassed or chased by other males.

#### Copulation call

**Figure 3:**
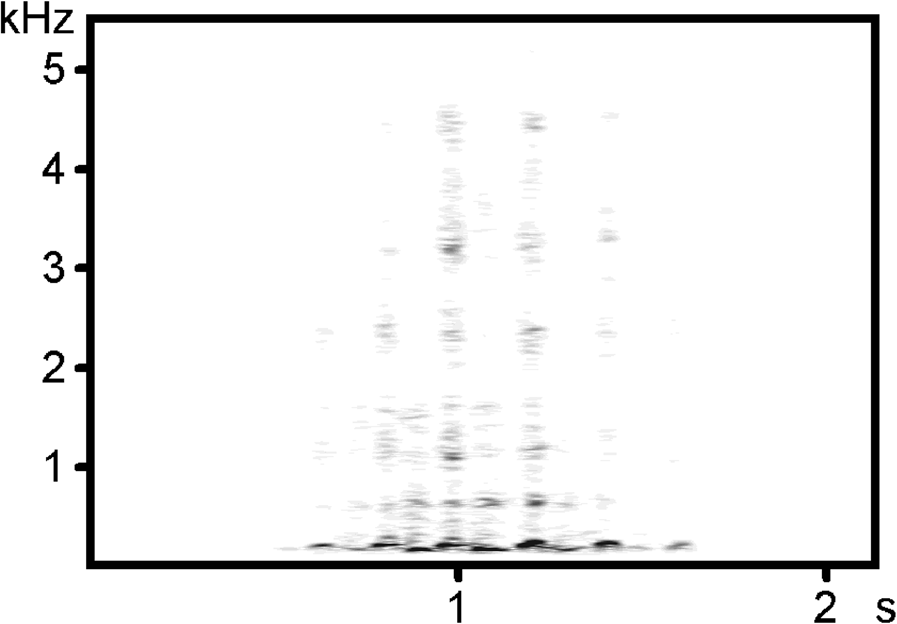
**Spectrogram of a sequence of a female copulation call.** The spectrogram was created by using Avisoft SASLabPro 5.1 (R. Specht, Berlin, Germany; fast Fourier transform resolution 1.024 points, sampling frequency: 5 kHz, time overlap: 96.87, Hanning window).

Copulation calls consisted of low amplitude, pant-like elements, the number of which varied substantially between as well as within individuals (Figure 3). Females produced these calls shortly after copulation, when they dashed from their copulation partner; however, these calls did not occur after each mating and were emitted rather infrequently. In total, we recorded only six calling events during the focal observations, and refrained from calculating the call rate.

#### Roar grunt

Roar grunts often occurred during or after aggression against females ( $\bar{x}$ = 83.3 ± 23.6%). Before calling, males usually ran up a tree and showed elements of display behaviour such as yawning and branch-shaking. Roar grunts were almost always produced in calling bouts (4 – 6 calls/bout). We further observed males uttering roar grunts during Forage/Travel contexts ( $\bar{x}$ = 11.0 ± 15.6%) when animals were widely scattered. Unlike reported for chacma baboons, roar grunts were not followed by series of long and protracted series of wahoo calls.

#### Grunt

Male as well as female grunts were the most common vocalizations. Grunts occurred mainly in non-agonistic, social contexts (Greeting, Infant handling and Affiliation). Only a minor proportion of grunts were produced during non-social contexts (‘Forage/Travel’ , males: $\bar{x}$ = 7.1 ± 3.4%, females: $\bar{x}$ = 11.2 ± 9.8%). Social grunts were almost always uttered in calling bouts with a varying number of calls per bout, contrary to non-social grunts which commonly occurred as single calls. The largest proportion of male grunts was produced when males engaged in affiliative interactions with females ( $\bar{x}$ = 39.3 ± 5.1%) as well as during infant handling ( $\bar{x}$ = 25.2 ± 4.5%). The remaining percentage of male grunts occurred during greeting interactions with other males ( $\bar{x}$ = 28.4 ± 9.5%). Females mostly grunted when interacting affiliatively with other females ( $\bar{x}$ = 48.1 ± 18.9%), as well as during infant handling ( $\bar{x}$ = 20.2 ± 8.5%) and during greeting with adult males ( $\bar{x}$ = 20.5 ± 19.8%).

#### Call rate

To assess the call rate we calculated the rate of each call type respectively (screams were not distinguished in the field). While the call rate of agonistic calls (i.e. scream, roar grunt) was extremely low (*<*0.1/h/individual), loud calls were produced slightly more often (wahoo: $\bar{x}$ = 0.14 ± 0.16 calls/h/individual, bark: 0.32 ± 0.62 calls/h/individual). Grunts were emitted at a much higher rate ( $\bar{x}$ = 47.80 ± 30.12 calls/h/individual), especially during non-agonistic social interactions (see Call usage above), and constitute the most common vocalization. Males grunted significantly more often than females ( $\bar{x}$_*male*_ = 71.05 ± 18.54 calls/h/individual, $\bar{x}$_*female*_ = 18.74 ± 4.92 calls/h/individual; exact Mann–Whitney U-test, *T* = 36, *p* < 0.001). In Table 4 the call rates of the different call types are shown. Grunts were produced during the early morning (7:00–8:00), when individuals socialised around the sleeping trees ( $\bar{x}$ = 12.82 ± 8.76 calls/h/individual). Afterwards, from 8:00–10:00, the call rate decreased ( $\bar{x}$ = 6.13 ± 4.09 calls/h/individual), shortly after subgroups start to forage or to travel, and did not change much anymore during the late morning (09:00–11:00_,_$\bar{x}$ = 6.33 ± 3.47 calls/h/individual). In the evening (17:00–19:00) the grunt rate was similarly low ( $\bar{x}$ = 6.69 ± 5.42 calls/h/individual).

**Table 4:** Call rate (Mean ± SD) of the different call types for both sexes

**Sex**	**Scream**	**Grunt**	**Roar grunt**	**Bark**	**Wahoo**
Male	0.04 ± 0.10 calls/h	71.05 ± 18.54 calls/h	0.08 ± 0.18 calls/h	-	0.14 ± 0.16 calls/h
Female	0.08 ± 0.10 calls/h	18.74 ± 4.92 calls/h	-	0.32 ± 0.62 calls/h	-

### Vocal variation of grunts

#### Relationships with caller sex

Male- and female grunts differed most in pitch characteristics of the calls such as fundamental frequency (F0, *F* = 112.9, *p* < 0.001) and distribution of the frequency amplitudes (DFA2 mean, *F* = 46.6, *p* < 0.001) which was higher in females than in males; as well as in call noisiness which was higher in males than females (e.g. Noise, *F* = 60.4, *p* < 0.001). Call modulation also differed between the sexes and was stronger in males than females, although to a lesser extent (e.g. DFB1ch mean, *F* = 5.2, *p* = 0.034; PFtr mean, *F* = 6.2, *p* = 0.023; see Table 5).

**Table 5:** Differences in grunt characteristics between males and females

**Parameters**	**Female**	**Male**	***F***	***p***
Duration [ms]	173 ± 32	186 ± 34	4.6	0.046
DFA2 mean [Hz]	502 ± 105	379 ± 55	46.6	0.000
DFB1ch mean [Hz]	10 ± 7	8 ± 7	5.2	0.034
Range [Hz]	984 ± 379	927 ± 253	0.5	0.488
PFtr mean [Hz]	85 ± 52	72 ± 40	6.2	0.023
Cs mean	0.96 ± 0.02	0.97 ± 0.01	3.8	0.067
F0 mean [Hz]	103 ± 15	61 ± 6	112.9	0.000
Noise [%]	11 ± 2	31 ± 19	60.4	0.000
Hnr1	0.22 ± 0.5	0.29 ± 0.05	24.1	0.000

LMM-analyses have been conducted with caller sex as fixed factor and caller ID as random factor. Mean ± SD and adjusted *p* vales are shown (Simes correction for multiple testing).

#### Relationships with behavioural context

**Figure 4:**
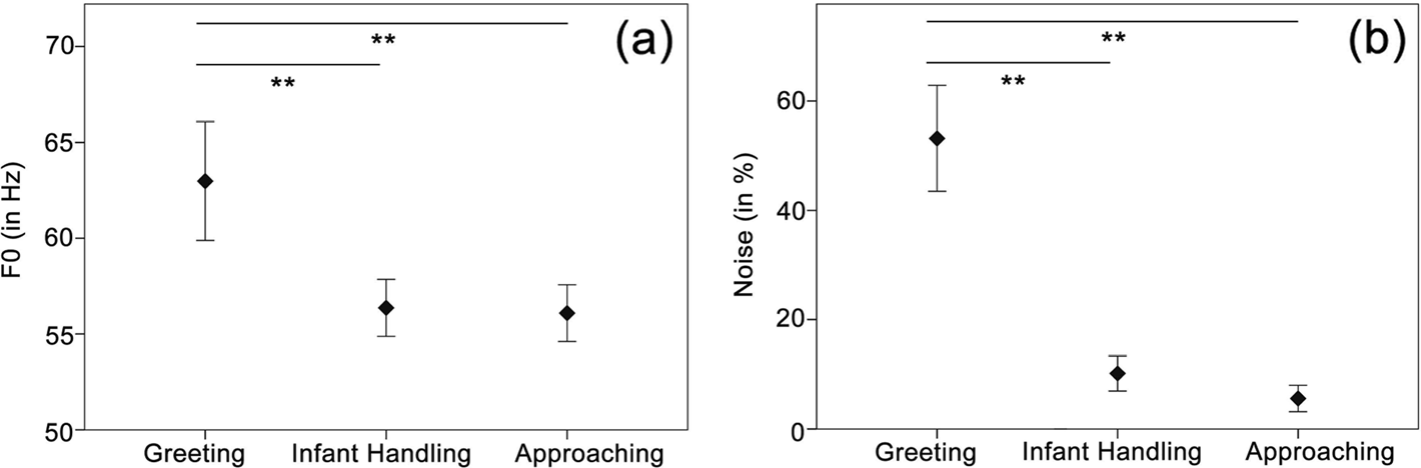
**Differences of (a) fundamental frequency (F0) and (b) call noisiness (Noise) between the social contexts.** Statistical significant differences (LSD Post-hoc test) only occurred between Greeting- and Infant handling/Affiliation context, but no differences were found between Infant handling and Affiliation. Vertical lines represent standard deviation. ** indicates statistical significance, *p* < 0.01 (*p* values were adjusted by using Simes correction for multiple testing).

For the analysis of context-related differences, we only compared calls produced in the three social contexts, since we did not have a sufficient amount of calls uttered during the Forage/Travel context. Grunts uttered in the three contexts differed most in terms of noisiness (Noise, *F* = 91.5, *p* < 0.001), followed by the fundamental frequency (F0, *F* = 16.1, *p* < 0.001), the modulation of the first frequency band (DFB1ch mean, *F* = 11.5, *p* < 0.001), and the harmonic to noise ratio (Hnr1, *F* = 11.2, *p* < 0.001). The results of the LMM for each acoustic parameter are shown in Table 6. An LSD post-hoc test conducted on those four parameters revealed significant differences only between the Greeting and Infant handling context (and between the Greeting and Affiliation context), but no statistical difference was found between Infant handling and Affiliation*.* The percentage of noise (Noise) in the calls was significantly higher in the Greeting context ( $\bar{x}$ = 53 ± 38%, *p* < 0.001) compared to both other contexts but was rather equally low during Infant handling ( $\bar{x}$ = 10 ± 15%) and Affiliation ( $\bar{x}$ = 5 ± 11%, *p* = 0.808). Similar results were found for the fundamental frequency (F0), which was higher during Greeting ( $\bar{x}$ = 62 ± 11, *p* < 0.001) compared to the Infant handling ( $\bar{x}$ = 55 ± 6) and Affiliation context ( $\bar{x}$ = 56 ± 7, *p* = 0.837). In Figure 4 the error bars of noise and fundamental frequency are illustrated for each context. The first frequency band was more strongly modulated (DFB1ch mean) during Greeting ( $\bar{x}$ = 6 ± 3, *p* < 0.001), than during Infant handling ( $\bar{x}$ = 2 ± 3) and Affiliation ( $\bar{x}$ = 2 ± 2, *p* = 0.165) and harmonic to noise (Hnr1) was lowest in the Greeting context ( $\bar{x}$ = 0.06 ± 0.04, *p* < 0.001) compared to the Infant handling- ( $\bar{x}$ = 0.10 ± 0.03) and Affiliation context ( $\bar{x}$ = 0.09 ± 0.03, *p* = 0.665).

**Table 6:** Variation in male grunt characteristics in three different contexts

**Acoustic parameters**	**Greeting**	**Infant handling**	**Affiliation**	***F***	***p***
Duration [ms]	289 ± 76	294 ± 219	229 ± 53	4.9	0.008
DFA2 mean [Hz]	400 ± 50	410 ± 42	396 ± 35	3.2	0.042
DFB1ch mean [Hz]	6 ± 3	2 ± 3	2 ± 2	11.5	0.000
Range [Hz]	741 ± 240	820 ± 293	896 ± 318	5.4	0.005
PFtr mean [Hz]	56 ± 34	53 ± 35	47 ± 35	1.3	0.262
Cs mean	0.99 ± 0.01	0.98 ± 0.01	0.99 ± 0.01	0.61	0.941
**F0 mean [Hz]**	**62 ± 11**	**55 ± 6**	**56 ± 7**	**16.1**	**0.000**
**Noise [%]**	**53 ± 39**	**10 ± 15**	**5 ± 11**	**91.5**	**0.000**
Hnr1	0.06 ± 0.04	0.10 ± 0.03	0.09 ± 0.03	11.2	0.000

Mean ± SD are shown and results of LMM analysis with context as fixed factor and caller ID as random factor, with adjusted *p* values (Simes correction for multiple testing). Bold numbers indicate the both most important parameters.

#### Relationships with body size

**Figure 5:**
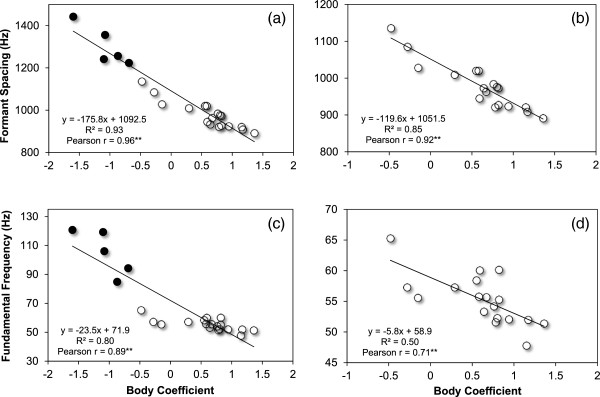
**Correlations between formant spacing (a, b) as well as fundamental frequency (c, d) and the calculated body coefficient (BC).** The left graphs shows the measures of all subjects (black dots indicating females, *N* = 5, and white dots indicating males, *N* = 18); the right graphs only show measures of males. BC is a compound measure of body size calculated from all body measurements by using principal component analysis. ^**^ indicates statistical significance, *p* < 0.01 (p values were adjusted by using Simes correction for multiple testing).

To investigate the relationship between the vocal structure of grunts and body size we conducted correlation analysis between the calculated body component (BC) and the fundamental frequency (F0) as well as formant spacing (∆*F*). Both F0 and (∆*F*) correlated significantly with body size. Animals with a higher BC uttered grunts with a lower fundamental frequency (*N* = 23, *r* = 0.89, *p* < 0.01) and smaller formant spacing (*N* = 23, *r* = 0.96, *p* < 0.01, see Figure 5). When the analysis was restricted to male calls only, formant spacing still correlated strongly with BC (*N* = 18, *r* = 0.92, *p* < 0.01), whereas the correlation between fundamental frequency and BC slightly decreased (*N* = 18, *r* = 0.71, *p* < 0.01); nevertheless, both correlation coefficients still showed a strong association between the vocal characteristics and individual body size. In Figure 5 (a-d) the scatter plots for formant spacing and fundamental frequency are shown.

## Discussion

The call types found in Guinea baboons, namely grunts, screams, barks, wahoos, roar grunts, and copulation calls, had all been described, either audio-visually or statistically, in several other baboon taxa (e.g. [16,62,74-78]). This lack of variation among the taxa is compatible with the assumption that evolutionary constraints play an important role limiting the flexibility in the structure of nonhuman primate vocalizations. Some of the differences between the values obtained here and those reported for other baboon taxa are most likely related to differences in body size. For instance, grunts of male- and female-Guinea baboons had a higher fundamental frequency than in chacma baboons [79]. The descriptions of other call types in baboons, such as loud calls [76], suggest a similar morphological pattern. However, for detailed inter-specific comparisons quantitative analyses, using similar acoustic parameters and standardized classification methods, among different baboon taxa are crucially needed.

The usage of the Guinea baboon vocal signals shows considerable differences from that of other baboon taxa. One of the most striking findings is males hardly ever emit loud calls during competitive interactions. Although agonistic interactions occur among male Guinea baboons, the competition is much less pronounced than in chacma baboons, for instance. In chacma baboons, males compete aggressively for social rank which confers priority of access to resources and, most importantly, fertile females [80]. Male loud call displays function as an important indicator of fighting ability [26,81], but they are also used to coerce females [82]. Males use loud call displays as a conflict management strategy to avoid the costs of fighting [26]. In Guinea baboons however, competition appears to be much less intense and male-male relationships lack obvious dominance hierarchies; thus, there is less pressure to settle conflicts using signals in order to avoid costly fighting [83]. The observation that competition in male Guinea baboons is reduced is, perhaps, also reflected by the diminished usage and structure of female copulation calls. In savannah baboons, they are uttered after almost each mating and consist of a rhythmic succession of high intensity, pant like calls [84,85], and they are believed to incite male competition for females [86]. Furthermore, formalized threats, an important dominance behaviour of female savannah baboons [73], only occurred occasionally in Guinea baboons.

While the usage of display vocalizations is greatly reduced, males grunt much more frequently than chacma baboons. These grunts occurred mainly during greetings or affiliative interactions among males, reflecting a higher rate of positive interactions [Patzelt A, IN, Fickenscher G, JF, unpublished data]. Particularly noteworthy is the high rate of greetings among males [65]. Greetings may be used not only to bond with other males, but also as “agonistic buffering” to reduce aggressive tendencies [87,88]. Males also grunt during affiliative interaction with females, and during infant handling, a pattern that can also be found in other baboon taxa [71]. Female Guinea baboons grunted mostly during affiliative interactions.

Differences in grunt structure between males and females are probably simply a reflection of the size dimorphism. Formant dispersion as well as fundamental frequency were higher in female compared to male grunts and strongly correlated with individual body size [89,90]. In addition, it may be that the sexes differ with regard to the affective state during calling [75,91]. Male grunts varied in relation to behavioural context; grunts uttered during male-male greetings were much harsher and noisier compared to non-greeting grunts, which may be related to a higher degree of arousal [91,92].

We did not find evidence that the vocal repertoire of Guinea baboons is more complex than that of other baboon taxa with a more stable and less complex social organization, refuting the idea of that variation in social organization has a rapid effect on vocal complexity. Between genera and over longer time scales, variation may of course evolve. For instance, a recent study reported that Gelada males (*Theropithecus gelada)* exhibit a higher diversity of call types than Chacma baboons [9]. Geladas also live in a multi-level, fission–fusion society. However, within their herds, they only interact with a small number of subjects that belong to the same reproductive unit [93]. Both Chacma baboons and geladas live in matrilineal groups with male dispersal [94,95]. The comparison of determinants of vocal complexity in geladas and the various members of the genus *Papio* highlight the need for greater clarification of the notion of “social complexity”. While the multi-level aggregation of geladas and Guinea baboons appears more complex at the level of the social organization, it remains to be quantified if their social relationships are more differentiated as well. Interestingly, it appears that gelada males focus their social attention on members of their own reproductive units [96], suggesting that at the level of the social relationships, their social life may be less complex than those of Chacma baboons. As for Guinea baboons, a playback study revealed that they keep track of the interactions of their gang members, while largely ignoring simulated intrusions by neighbors or strangers [97]. Whether or not they maintain more diverse and more differentiated relationship than members of the respective other groups remains an issue for empirical investigation.

Within the genus *Papio*, we do not deem habitat quality to be an important driver with regard to the morphology of the vocal repertoire, as there is more variation in environmental conditions within than between taxa [61]. Nevertheless, previous research has shown that short-term fluctuations in visibility may directly affect calling rates. When the visibility was poor and the risk of losing contact with the social partners increased, olive baboons females called more frequently than in open habitats [98,99].

## Conclusions

In summary, our findings support the view that the basic structure of the sound patterns – presumably at the level of the motor pattern generators in the lower brain stem – is relatively similar in different baboon taxa. This may either be explained by evolutionary constraints preventing higher rates of evolution, or a lack of selective pressures to evolve a higher diversity of calls. The observed variation in call structure between different taxa can probably be explained by variation in body size and vocal tract morphology. Nevertheless, we do find some noteworthy variation in the degree of expression of different vocal patterns, such as the absence of pronounced “hoo-syllables” in male wahoos, and the low amplitude of copulation calls, if they are produced at all. The greatest degree of flexibility, finally, can be found in call usage, where the occurrence of either more affiliative or more competitive relationships drives the usage of the corresponding call types. Our results do not lend support to the assumption that an increased complexity at the level of the social organisation necessarily leads to higher vocal complexity. Instead, competition and cooperation more specifically affect the use of vocal signals to regulate social relationships.

## Methods

### Study site

Research took place at the field station of the German Primate Center (DPZ), the Centre de Recherche de Primatologie (CRP), located in the Simenti region of the Niokolo Koba National Park (13°01′34′´N, 13°17′41‘W). The park lies across the borders between Senegal-Oriental and La Casamance close to the Guinean border in southeast Senegal and covers an area of more than 910,000 ha. The climate is of a Sudanian type with a dry season from November until June and a rainy season from July until October. The rainfall during the study period added up to 124 mm during the dry season and to 885 mm during the rainy season. The mean minimal temperature was 24.0 and 25.4°C and the maximum mean temperature 36.9 and 32.3°C in the dry season and in the rainy season, respectively (Simenti weather station, measured for 2010). The whole area has superficial formations of laterite and sediments and is watered by several ponds and large waterways, such as the Gambia and Niokolo Rivers. The vegetation varies from a southern Sudanian type to a Guinean savannah type and comprises gallery forests (close to the river banks), seasonally flooded grassland and dry deciduous forest. There are about 80 mammal species, 330 bird species, 36 reptiles and 20 species of amphibians recorded in the park and, despite a dramatic decrease in large mammal population sizes during the last decades, potential predators such as lions (*Panthera leo*), leopards (*Panthera pardus*) and spotted hyenas (*Crocuta crocuta*) still exist in this region [100].

### Subjects

Since 2010 two Guinea baboon gangs are fully- and two gangs semi-habituated to human observers (Mare gang = M, Simenti gang = S, River gang = R, N = Nose gang, i.e. around 200 individuals). The size and composition of the four study gangs varied considerably and comprised approximately 50–55 individuals in gang M, 55–60 individuals in gang S, 25–35 individuals in Gang R and 20–30 individuals in gang N. To track the whereabouts of the focal gangs, two males in each gang were fitted with radio collars. Furthermore, 12 individuals from three different gangs (gang M, gang S and gang R) were fitted with GPS collars, taking GPS fixes every two hours during the day and every three hours during the night. Based on the GPS fixes we assessed the home range of the study community to be ~ 36 km^2^ (ArcGIS 2010, ESRI Inc., Redlands, US).

### Ethical statement

The study was approved by the Diréction des Parcs Nationaux and the Ministère de l′Environnement et de la Protéction de la Nature de la République du Sénégal (Permit numbers: 0383/24/03/2009; 0373/10/3/2012). All capturing and handling procedures were carried out in accordance with the recommendations of the animal welfare deputy of the DPZ and the conservation authorities of the Diréction des Parcs Nationaux du Senegal. They complied with the current law of Germany and Senegal and were either conducted or accompanied by veterinaries of the Diréction des Parcs Nationaux du Senegal. All measurements were performed under anaesthesia and all efforts were made to prevent suffering during and after the procedure (see below for the detailed methods). Guinea baboons do not depict a protected species and are listed as near threatened by the IUCN (IUCN ver 3.1, 2008).

### Vocal repertoire

We collected data over 12 months distributed over two dry seasons (January-July 2010 and February-July 2011). Data collection began on the early morning hours, at 07:00 and proceeded until 12:00, as well as on the late afternoon from 17:00 until 19:00 UTC. We recorded vocalisation during *ad libitum* and focal animal sampling. For each audio-recorded vocalization we noted time/date, call type (see Results), the identity of the caller, the behavioural context, if possible the call receiver, individuals in caller proximity as well as the height of the caller (when sitting in a tree) and the recording distance. We defined six broad behavioural contexts, two non-social and four social ones, based on the callers’ behaviour (non-social contexts: ‘Forage/Travel’ , ‘Alarm’; social contexts: ‘Agonistic’ , ‘Greeting’ , ‘Affiliation’ (includes friendly approach and grooming each other) and ‘Infant handling’). The potential call receiver was identified by the orientating behaviour of the caller during calling (looking, approaching toward- and/or interacting with the individual). Calls were recorded using a digital solid-state recorder (Marantz PMD 661, Marantz, Kanagawa, Japan) and a Sennheiser directional microphone (K6 power module and a ME66 recording head with a Rycote softie windscreen; Sennheiser Electronic KG, Barleben, Germany) with a sampling frequency of 44.100 Hz, 16-bit resolution and the double-mono setting.

### Acoustic analyses (repertoire)

**Figure 6:**
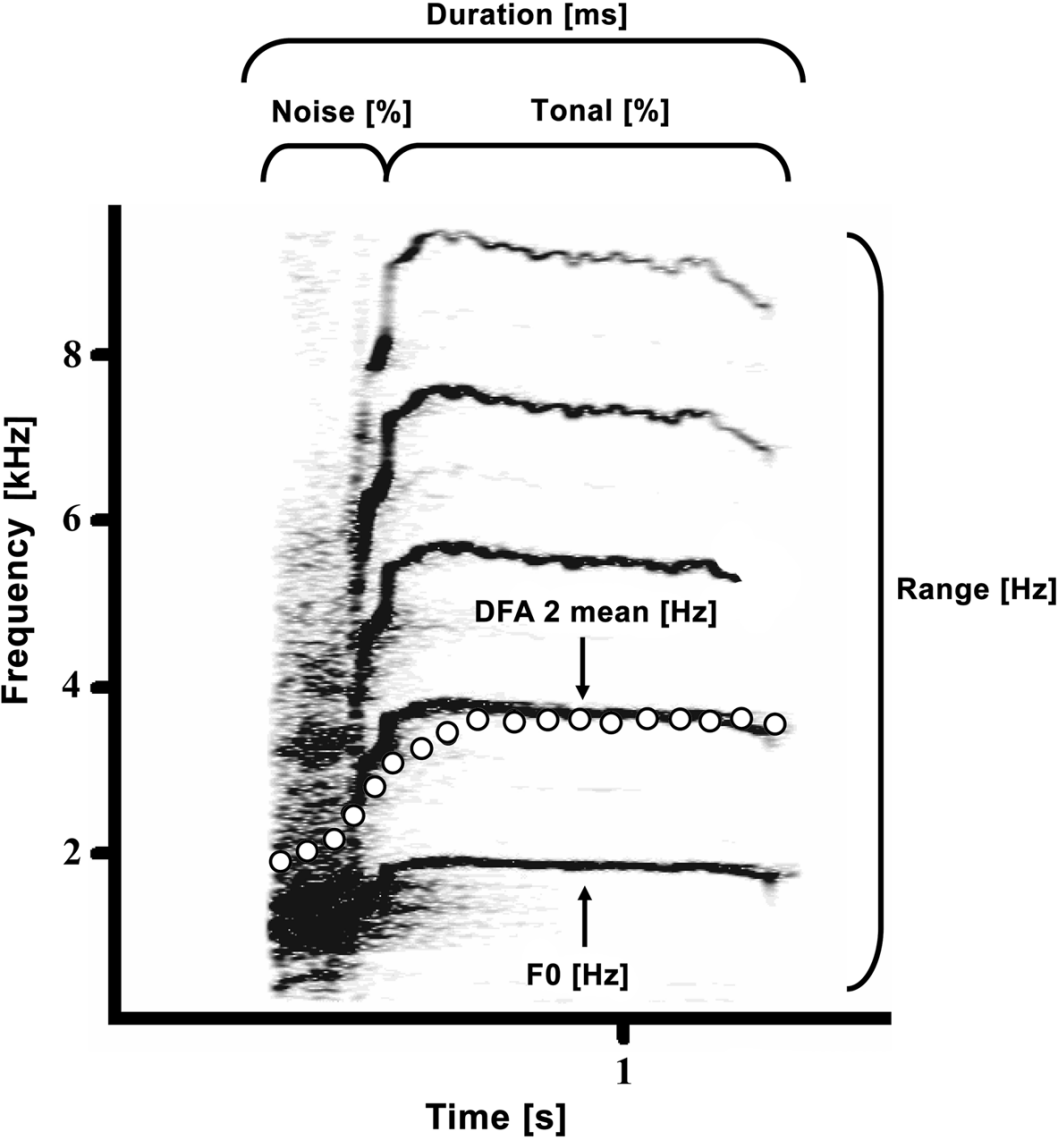
**Spectrogram of a female Guinea baboon call.** Five out of the nine call parameters used in the acoustic analyses are indicated in the spectrogram. White circles mark the DFA 2 (Fast Fourier transform-length 1024-points, Hann window, overlap 97.75%, time resolution 3.2 ms).

We recorded a total of 4420 calls. Since calls can be strongly distorted over longer distances, we only analysed calls recorded between 3–10 m. We used the software Avisoft-SAS Lab Pro 5.2 (R. Specht, Berlin, Germany) to select high quality calls. Calls which could not be clearly assigned to an individual or which were disturbed by background noise were excluded from the analyses. In total 1215 calls were used for the acoustic analyses. We audio-visually pre-classifed the various call structures in screams, barks, wahoos, roar grunts and grunts (see Results). To obtain a balanced distribution of the different call types we randomly selected 8–12 calls from each pre-classified call structure from 18 males and 12 females. Due to the large frequency range of the different call structures we adjusted the sampling frequencies accordingly: grunts and roar grunts to 5000 Hz and barks, wahoos, screams to 20000 Hz, resulting in a frequency range of 2500 Hz and 10000 Hz. After cutting the selected calls we saved the binary spectrogram (fast Fourier transform-length: 1024-points, Hanning window, overlap 96.4%) and exported them in the acoustic analysis software LMA 2012 [101]. To determine the fundamental frequency (F0) in low pitched grunt-calls we further lowered the sampling frequency to 1200 Hz and exported the binary spectrogram into LMA 2012 (fast Fourier transform-length 1024 points, Hanning window, overlap 98.9%). For all acoustic analysis we chose a set of nine acoustic parameters that broadly describe the temporal- and spectral characteristics of the vocalizations as well as the call tonality and the spectral modulation of the calls (see Table 1). The call morphology of the different call types could be sufficiently described with this set of acoustic parameters and a higher number of parameters did not have any advantage for the cluster analyses, as highly correlating acoustic parameters rendered the identification of appropriate cluster centers difficult. LMA was used to extract the acoustic parameters. We calculated the duration of the call (Duration), the statistical distribution of the frequency amplitudes (DFA 2 mean), the modulation of the first dominant frequency band (DFB1 mean), the overall frequency range (Range mean), the trend of the peak frequency (Pftr mean), the call consistency (Cs mean). Furthermore, tonal parameter such as the call noisiness (Noise), the harmonic to noise ratio (Hnr 1st) and the fundamental frequency (F0) were calculated. The F0 was assessed by using the manual tonality macro of LMA which is based on an autocorrelation function. This function only considers tonal elements of a call to calculate the fundamental frequency whereas noisy elements are ignored. The possible F0 range was set by visual adjustment of a harmonic cursor. Harmonic cursor of indicator lines spaced as multiple integer of the first (bottom) line. In this way they can help to detect visually periodic structures (tonal structures) in a call [102]. The F0 itself was estimated by an algorithm searching the highest frequency amplitude within the range of the lowest indictor. Figure 6 illustrates six acoustic parameters used for the analyses. The cut off frequency was set at 50 Hz to reduce background noise. The start and end thresholds were set at 15% and 10% for the calculation, which means that all time segments with a value lower than 15% of the maximal amplitude at the beginning and 10% at the end of the call were not considered. Cut of frequency as well as start- and end threshold were kept consistent for all call types.

### Call rate and call usage

To examine the call rate and the call usage we conducted 15 min all occurrences focal observations on 10 adult males and eight adult females in two gangs (gangs M and S). For each vocalization we noted date/time, caller ID, call type (see Results), behavioural context, habitat visibility and if applicable and possible, the call receiver. Focal samples were balanced over the day and the study period. In total we collected 190 hours of focal observation ( $\bar{x}$ = 10.2 ± 0.3 h/individual, $\bar{x}$ represent mean values ± SD). For each focal animal we calculated the call rate for each call type. In case of calling bouts (sequences of the same call type) we counted every call within the bout. The context specific call usage was calculated for all call types by dividing the number of calls uttered in each context by the total number of emitted calls of the respective call type.

To analyse sex-differences we compared male and female grunts (8–10 calls/individual, *N*_*male*_ = 10, *N*_*female*_ = 10) with each other. To check for context dependent differences we only analysed male grunts, since we had a sufficient number of calls produced in the different behavioural contexts only for males (6–10 calls/context/individual, *N*_*male*_ = 8).

### Anatomical measures

During regular trapping sessions we took body measurements. Animals were lured into individual cages (100 × 100 × 100 cm) with food. A vertically sliding door (50 × 50 cm) was closed manually by pulling a string (30–50 m) from a hide. When the other group members had left the area, we anaesthetized the subjects using 500 mg Xylacin + 4 ml Ketamin solution [10%] applied with a blowpipe. We measured the individuals with a standard commercial measuring tape and weighed them with a hanging scale. The length and width of the snout and the skull were measured with a vernier caliper. During the whole process we regularly controlled the body temperature, respiration and the corneal reflexes. The head was covered with a cloth, and the cornea was continuously wetted with medical tear supplement. For six individuals body measured were repeated two times in a row, revealing a rather moderate measurement error: $\bar{x}$ = 3.7 ± 5.1%. After the procedure we released the animals at the trapping site and guarded them until they fully recovered and walked off to join their group.

For statistical analysis, we applied a principal component analyses (PCA) to extract a single compound body measurement. Table 7 shows the body measures and their loadings on the body coefficient (BC). To investigate the influence of the body size on the call structure we analysed grunts and extracted one source- (fundamental frequency) and one filter- (formant spacing) related acoustic feature. Formants were measured by linear predictive coding (LPC) using Avisoft-SAS Lab Pro Recorder 5.2 (Hann window, 15 LPC coefficients). To determine formant spacing it is essential to analyse high quality calls with at least three clearly detectable formants (*F1* – *F3*). Only a limited number of calls fulfilled this criterion, hence, after visual inspecting our calls merely 3–5 calls per individual were suitable to analyse (*N*_*male*_ = 18, *N*_*female*_ = 5). We derived the formant spacing (∆*F*) from the frequencies of the first three formants by finding the best fit to the equation

$$F_{i}\;=\;\frac{2i-1}{2}\;\mathrm{\Delta F}$$

which relates individual formant frequencies to average overall format spacing in the vocal tract, approximated as a uniform tube closed at one end (the glottis) and open at the other (the mouth). A detail description of this procedure is given in [103].

**Table 7:** Body measurements of males and females

**Body measurement**	**Male**	**Female**	**Correlation with BC**
Chest circumference [cm]	57.9 ± 4.4	45.9 ± 3.7	0.94
Waistline [cm]	45.7 ± 2.1	38.8 ± 1.4	0.89
Arm length [cm]	53.4 ± 2.4	47.4 ± 2.5	0.90
Leg length [cm]	47.0 ± 2.5	39.7 ± 1.9	0.92
Skull length [cm]	10.9 ± 0.6	9.4 ± 0.7	0.62
Skull width [cm]	10.9 ± 0.8	9.1 ± 0.7	0.83
Snout length [cm]	9.9 ± 0.6	6.7 ± 0.5	0.88
Snout width [cm]	4.2 ± 0.4	3.2 ± 0.3	0.88
Back length [cm]	45.2 ± 2.9	39.6 ± 5.2	0.77
Body length [cm]	56.6 ± 3.2	51.1 ± 6.9	0.81
Weight [kg]	20.5 ± 2.1	11.4 ± 1.8	0.96

Mean ± SD (*N*_*male*_ = 18, *N*_*female*_ = 5) and respective factor loadings on the body coefficient (BC) are shown.

### Statistical analyses

To statistically describe the vocal repertoire we used a two-step cluster analyses on the selected acoustic variables, which has been already successfully applied in other bioacoustic studies [42,104]. We used the log-likelihood distance measure and the Schwarzsches’ Bayes cluster criterion (BIC) to calculate different clusters solutions. In addition, we qualitatively assessed the cluster solution by inspecting the silhouette values [105]. The silhouette value (*Sc*) represents the summarized distance of all within-cluster data points (*a*_*i*_) subtracted from the summarized distance to the data points of the successive cluster (*b*_*i*_) and finally divided by the sum of the larger distance:

$$S\left(c\right)\;=\;\frac{b\left(i\right)\;-\;a\left(i\right)}{\text{max}\left\{a\left(i\right)\;,\;b\left(i\right)\right\}}$$

Subsequently, the average value across all call clusters of the respective solution is calculated and gives a number between −1.0 and 1.0; cluster solutions with a *Sc* exceeding 0.5 are usually considered to be solid [105]. Hence, we calculated a set of different cluster solutions (2–14 clusters) and extracted the *Sc* for each solution. We further compared the formal categorization results to the audio-visual pre-classification of the calls and calculated the percentage of accordance between both classifications.

Afterwards, we ran a discriminant function analyses (DFA, SPSS 20) with the same acoustic parameters and the calculated call-clusters as grouping variable to evaluate the selected cluster solution and to estimate how the acoustic parameters contribute to the classification. We used a stepwise DFA and the assignment of calls was cross-validated by the leaving-one-out method of SPSS 20.

To analyse sex and context differences of grunts we carried out a linear mixed model analysis (LMM) on the same acoustic variables as used for the cluster analysis, with animal ID as random factor and sex and context as fixed factors, respectively. To identify significant differences between the contexts we applied univariate least significant differences (LSD) post-hoc tests. The influence of body size on the call structure we investigated by conducting a Pearson correlation analysis between the body component and fundamental frequency as well as formant spacing. All statistical tests were two-tailed and conducted with SPSS 20 or the statistical package R (R Development Core Team). We corrected for multiple testing by adjusting all *p*-values using Simes correction.

## Competing interests

The authors declare that they have no competing interests.

## Authors’ contributions

PM recorded the data, conducted the analyses and wrote the paper, IN did the veterinary work in the field and collected body measurements, KH designed the study and contributed to the acoustic and statistical analyses and assisted by drafting the paper, JF designed the study and wrote the paper. All authors read and approved the final manuscript.
